# Analysis of health funding and expenditure in the municipalities of Sergipe state, Brazil: an ecological study, 2005-2020

**DOI:** 10.1590/S2237-96222025v34e20240107.en

**Published:** 2025-05-12

**Authors:** Millena Barroso Oliveira, Vinícius Henrique Ferreira Pereira de Oliveira, Cauane Blumenberg, Douglas Teixeira da Silva, Álex Moreira Herval, Luiz Renato Paranhos

**Affiliations:** 1 Universidade Federal de Uberlândia, Faculdade de Odontologia, Programa de Pós-Graduação em Odontologia, Uberlândia, MG, Brazil; 2 Universidade Estadual Paulista, Faculdade de Odontologia, Programa de Pós-Graduação em Odontologia, Araraquara, SP, Brazil; 3 Universidade Federal de Pelotas, Faculdade de Medicina, Programa de Pós-Graduação em Epidemiologia, Pelotas, RS, Brazil; 4 Universidade Federal de Uberlândia, Faculdade de Odontologia, Área de Odontologia Preventiva e Social, Uberlândia, MG, Brazil

**Keywords:** Health System Financing, Health Investments, Health Expenditures, Public Expenditures on Health, Ecological Studies, Financiación de los Sistemas de Salud, Inversiones en Salud, Gastos en Salud, Gasto Público en Salud, Estudios Ecológicos

## Abstract

**Objective:**

To analyze part of health funding and expenditure in the municipalities of Sergipe state.

**Methods:**

This is an epidemiological, observational, ecological study, using Sergipe public health budget data, for the period 2005-2020. The units of analysis were the state’s municipalities. The data were collected from the Public Health Budget Information System, based on the following indicators: municipal revenue from Brazilian National Health System funding transfers, municipality funded expenditure and total expenditure on health. Absolute average annual variance in revenue and expenditure indicators were estimated using linear least squares models weighted for variance.

**Results:**

Data for the 75 municipalities of Sergipe were assessed. The analyses showed that, in 2020, the lowest amount of health transfer revenue was BRL 244.00 per inhabitant, transferred to São Cristóvão. The highest amount was BRL 743.00 per inhabitant, allocated to São Francisco. Health transfer revenue increased in all municipalities studied over the years analyzed, with an absolute average annual increase of BRL 23.00 per inhabitant.

**Conclusion:**

There was an absolute average annual increase in health transfer revenue allocated to municipalities. Transfer was uneven among the municipalities analyzed.

## Resumen

**Objetivo:**

Analizar parte del financiamiento y gasto en salud en los municipios del estado de Sergipe.

**Métodos:**

Se trata de un estudio epidemiológico, observacional, ecológico, utilizando datos del presupuesto de salud pública de Sergipe, para el período 2005-2020. Las unidades de análisis fueron los municipios del estado. Los datos fueron recolectados del Sistema de Información sobre Presupuestos Públicos en Salud, basados en los indicadores: ingresos por transferencias a la salud – Sistema Único de Salud, gasto con recursos propios y gasto total en salud. Las variaciones anuales promedio absolutas de los indicadores de ingresos y gastos se estimaron utilizando modelos lineales de mínimos cuadrados ponderados por la varianza.

**Resultados:**

Se evaluaron datos de 75 municipios de Sergipe. Los análisis mostraron que, en 2020, el valor más bajo de ingresos por transferencias de salud fue de BRL 244,00 por habitante, transferidos a São Cristóvão. El valor más alto fue de BRL 743,00 por habitante, destinado a São Francisco. Los ingresos por transferencias de salud aumentaron en todos los municipios estudiados a lo largo de los años analizados, con un aumento anual promedio absoluto de BRL 23,00 por habitante.

**Conclusión:**

Hubo un aumento anual promedio absoluto en los ingresos provenientes de las transferencias de salud asignadas a los municipios. La transferencia fue desigual entre los municipios analizados.

## Introduction

The Brazilian National Health System (*Sistema Único de Saúde* - SUS) is recognized as one of the largest public health systems in the world, ensuring universal access to health actions and services (1-3). To achieve these objectives, funding is based on a tripartite model, in which the federal, state and municipal governments have minimum percentages of investment in health (4-6). However, budgetary constraints and inequalities in the distribution of resources compromise the equitable implementation of the system (7). Systematic monitoring of the sources and applications of financial resources effectively invested in health is important (8). Studies show that the transfer of federal resources for health services is unevenly distributed among states and municipalities, creating challenges in the funding of Primary Health Care (9,10).

Analysis of health funding and expenditure in the Brazilian state capital cities revealed that, between 2008 and 2018, the transfer of federal resources for health services showed an uneven distribution pattern between these cities (11). The analyses showed that, in 24 of the capitals studied, there was a significant annual increase in health revenues obtained from the transfer of federal resources. Aracaju (capital of the state of Sergipe) was an exception, as it presented a statistically constant amount of federal resource transfers throughout the years analyzed (11).

The scarcity of detailed analyses on funding trends in the state of Sergipe justifies the need to investigate the behavior of financial resources allocated for health services in the municipalities of Sergipe. This study aims to analyze part of health funding and expenditure in the municipalities of Sergipe.

## Methods

### Design and period

This is an epidemiological, observational, ecological study. We followed the REporting of Studies Conducted using Observational Routinely-collected health Data (12) statement as a basis for reporting on our study.

We used Sergipe public health budget data for the period 2005-2020. The 15-year period was chosen to permit comprehensive longitudinal analysis, enabling observation of trends and variations in expenditure and revenue over the years assessed. The units of analysis were the state’s municipalities.

### Setting

**Figure 1:**
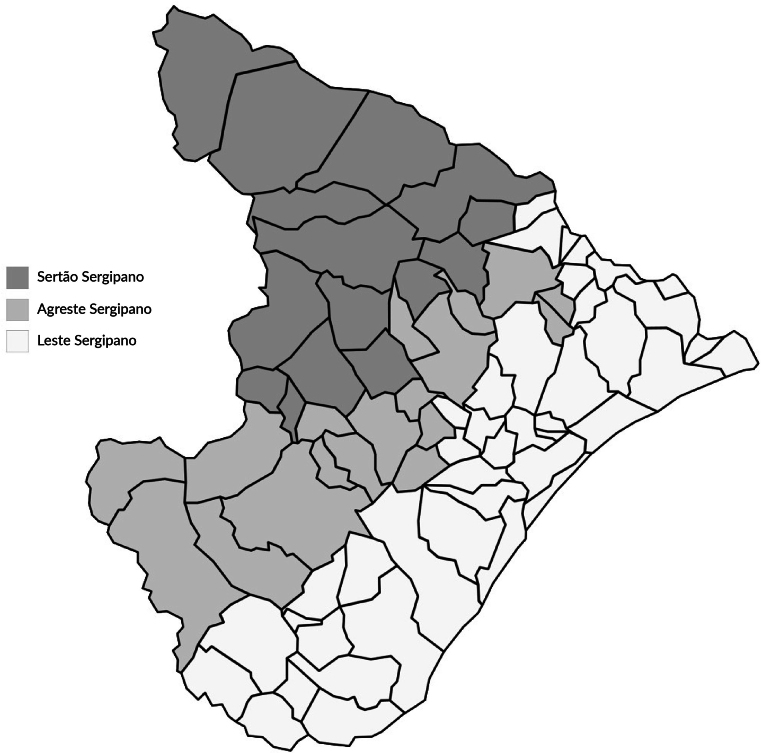
Municipalities of Sergipe, grouped together according to the state’s three mesoregions. Sergipe, 2024

Located in the Northeast region of Brazil, the state of Sergipe has a territorial area of ​​21,938.188 km², bordered to the east by the Atlantic Ocean, to the west and south by the state of Bahia, and to the north by the state of Alagoas (Figure 1) (13). Sergipe has an estimated population of 2,210,004 inhabitants. The smallest municipality in terms of population in 2020 was Amparo de São Francisco, with 2,374 inhabitants, while Aracaju (the state capital) was the largest, with 657,013 inhabitants. Sergipe has 1,115 health establishments, of which 790 are public and 325 are private. Of these, 921 provide SUS services, totaling 884 beds for hospitalization (13).

### Variables

The following indicators were analyzed: i) Brazilian National Health System (SUS) funding transfer revenue (*receita das transferências para a saúde* – SUS: “R. Transf. SUS”), which includes transfers from the Ministry of Health for expenditure in local health services (8); ii) municipality funded expenditure (*despesa com recursos próprios*: “D. R. Próprios”), which refers to the total health expenditure incurred by municipalities (8); and iii) total health expenditure (*despesa total com saúde*: “*D. Total Saúde*”), which represents total expenditure on health actions and services, funded by municipalities, states and the federal government (8).

### Data sources and measurement

The public health budget data for the municipalities of Sergipe were obtained through the Public Health Budget Information System (*Sistema de Informações sobre Orçamentos Públicos em Saúde* - SIOPS), which is operated by the Ministry of Health via its SUS Information Technology Department (14). The data available on the SIOPS platform and must be reported by all government health departments. The indicators were collected as gross values ​​in BRL for each year of interest. 

In order to collect the data, we accessed the SIOPS website at https://www.gov.br/saude/pt-br/acesso-ainformacao/siops (14). We selected the following icons: “Indicators” (“Indicadores”) and “Indicator Time Series - Municipalities” (*Série Histórica de Indicadores* - Municípios). We then selected “Sergipe” on the “Municipal Indicators” (“Indicadores Municipais”) page. On the following page, data were collected by selecting the indicators “R. Transf. SUS”, “D. R. Próprios” and “*D. Total Saúde*”, between 2005 and 2020. Using the “Municipality” (“Município”) tab, we selected the option “All categories” (“Todas as categorias”). All data used in the study were obtained on January 24, 2022.

### Deflationing

Variance in revenue and expenditure over the years may occur simply due to inflation, which may compromise comparison of amounts ​​over different periods. In order to correct this problem and enable comparison of revenue and expenditure between different years, the amounts ​​were deflated based on the 2020 Broad National Consumer Price Index (Índice Nacional de Preços ao Consumidor Amplo) (15), this being the last year considered in the analysis.

The following formula was used for deflationing:

*True amount* = *Nominal Amount* × *Price Index*

where the nominal amount was the amount as at 2005 for each of the three indicators assessed. 

After being deflated, the amounts ​​were used to calculate the ratio of revenue and expenditure in relation to the resident population in the municipalities for each year – generating estimates per inhabitant. 

### Statistical methods

The first step of the statistical analysis consisted of assessing the covariance structure of the outcomes of interest. Linear regression models containing the outcome of interest and the year as a predictor were adjusted for each municipality in Sergipe. The Breusch-Pagan test (with Bonferroni adjustment for multiple comparisons) was used to assess the variance structure and the possibility of heteroscedasticity. Nine percent of the models indicated presence of heteroscedasticity. We chose to use linear least squares models weighted for variance to estimate average absolute annual variance in SUS funding transfer revenue per inhabitant, municipality funded expenditure per inhabitant and total health expenditure per inhabitant. These methods are robust in situations of heteroscedasticity and allow for adequate estimation of the regression coefficients, controlling for autocorrelation of revenue and expenditure amounts ​​over the years.

The coefficient generated by this regression analysis can be interpreted as the absolute annual average variance of the indicator being assessed, indicating the annual trend of revenue and expenditure indicators between 2005 and 2020. Three regression models were adjusted, one for each revenue and expenditure indicator. In the models, the indicators were included as the outcome, while the year (2005-2020) was considered as the main predictor. All analyses used a 5% significance level and were performed using Stata 17 software (StataCorp LLC., College Station, Texas), using the vwls command. Statistical significance was assessed based on the overlap of estimates and 95.0% confidence intervals.

## Results

We assessed all 75 municipalities of Sergipe. SUS funding transfer revenue per capita in 2020 ranged from BRL 743.00 per inhabitant in São Francisco to BRL 244.00 per inhabitant in São Cristóvão. SUS funding transfer revenue increased in all 75 municipalities analyzed, with an average annual increase of BRL 23.00 per inhabitant. The highest average annual increase was BRL 40.00 per inhabitant per year, recorded in Malhada dos Bois. The lowest average annual increase was BRL 9.00 per inhabitant per year, found for São Cristóvão (Table 1).

**Table 1:** Absolute average annual variance and 95% confidence intervals (95%CI) of municipal revenue from Brazilian National Health System funding transfers per inhabitant, municipality funded expenditure per inhabitant and total expenditure on health per inhabitant, by municipality. Sergipe, 2005 and 2020

Municipality	Population in 2020	Municipal revenue from Brazilian National Health System funding transfers (BRL)			Municipality funded expenditure (BRL)			Total health expenditure (BRL)		
		2005	2020	Variance (95%CI)	2005	2020	Variance (95%CI)	2005	2020	Variance (95%CI)
Aracaju	657,013	194	478	15 (4; 26)	70	407	25 (16; 35)	235	875	42 (22; 62)
Nossa Senhora do Socorro	183,628	60	288	12 (5; 20)	43	285	13 (7; 20)	103	543	24 (13; 36)
Lagarto	104,408	84	359	14 (8; 20)	27	162	10 (6; 14)	108	466	23 (15; 32)
Itabaiana	95,427	86	539	26 (15; 37)	27	187	11 (7; 15)	106	747	38 (24; 52)
São Cristóvão	90,072	30	244	9 (5; 14)	31	180	9 (5; 13)	60	414	19 (11; 27)
Estância	69,184	96	469	24 (12; 36)	59	244	14 (8; 21)	155	672	36 (20; 52)
Tobias Barreto	52,191	35	296	14 (8; 20)	47	171	8 (3; 13)	82	420	22 (12; 32)
Itabaianinha	41,928	33	404	34 (6; 62)	23	162	9 (5; 13)	55	525	31 (20; 43)
Simão Dias	40,484	26	332	20 (13; 28)	43	186	10 (6; 14)	61	522	32 (19; 44)
Nossa Senhora da Glória	36,924	37	377	22 (11; 33)	37	213	12 (7; 16)	74	644	31 (18; 44)
Poço Redondo	34,775	39	300	17 (10; 24)	63	168	10 (5; 14)	95	336	26 (13; 38)
Itaporanga d’Ajuda	34,356	30	442	24 (15; 33)	56	390	21 (13; 28)	86	824	44 (29; 60)
Capela	34,213	36	402	20 (12; 28)	36	265	11 (5; 17)	71	618	28 (16; 39)
Barra dos Coqueiros	30,407	45	288	12 (8; 17)	82	881	47 (31; 64)	116	1,080	60 (40; 80)
Canindé de São Francisco	29,900	67	412	24 (13; 34)	144	430	6 (-4; 16)	216	879	33 (16; 50)
Laranjeiras	29,826	49	426	21 (13; 30)	136	271	19 (4; 35)	184	692	40 (22; 59)
Propriá	29,626	49	576	34 (22; 47)	39	199	11 (6; 16)	87	804	46 (32; 59)
Porto da Folha	28,596	33	436	26 (16; 36)	38	192	10 (6; 14)	71	683	35 (23; 47)
Boquim	26,816	33	410	21 (12; 29)	49	178	9 (5; 13)	83	538	28 (17; 40)
Nossa Senhora da Dores	26,629	50	554	29 (17; 40)	39	236	16 (7; 25)	89	924	49 (30; 68)
Umbaúba	25,294	35	278	15 (8; 23)	49	241	13 (7; 19)	86	514	27 (16; 38)
Poço Verde	23,728	36	315	17 (10; 24)	55	204	11 (5; 17)	98	600	30 (17; 43)
Carira	22,082	28	393	20 (13; 28)	47	214	10 (5; 15)	76	542	30 (19; 40)
Aquidabã	21,563	46	422	21 (13; 30)	47	201	10 (5; 15)	77	635	34 (21; 46)
Salgado	19,998	29	320	23 (14; 32)	68	194	9 (5; 13)	85	552	27 (17; 38)
Riachão do Dantas	19,805	36	358	19 (12; 26)	40	208	8 (3; 12)	71	578	26 (16; 36)
Campo do Brito	18,109	40	387	24 (13; 34)	51	215	13 (5; 22)	84	666	36 (23; 50)
Japaratuba	18,743	55	449	23 (10; 37)	138	432	17 (6; 28)	184	945	44 (22; 65)
Neópolis	18,719	40	354	22 (13; 31)	58	299	15 (9; 22)	95	691	42 (26; 58)
Ribeiropólis	18,652	46	466	23 (15; 32)	58	248	14 (8; 21)	96	735	35 (23; 47)
Areia Branca	18,542	36	400	18 (10; 27)	55	377	22 (12; 32)	94	798	38 (22; 55)
Indiaroba	17,957	49	399	17 (8; 25)	68	249	12 (6; 18)	113	626	33 (18; 48)
Cristinápolis	17,874	37	490	27 (18; 37)	46	274	18 (10; 25)	83	802	45 (31; 59)
Maruim	17,213	55	409	25 (16; 33)	83	394	23 (14; 33)	142	886	49 (32; 67)
Carmópolis	16,634	26	288	16 (9; 24)	162	764	25 (11; 40)	178	1,161	53 (34; 71)
Frei Paulo	15,421	45	416	22 (13; 32)	54	235	13 (6; 20)	100	737	35 (21; 48)
Monte Alegre de Sergipe	15,031	47	379	19 (9; 28)	54	316	16 (11; 21)	99	601	31 (19; 43)
Pacatuba	14,428	57	366	24 (15; 32)	114	361	19 (10; 28)	168	710	38 (24; 53)
Santa Luzia do Itanhy	14,035	36	353	19 (9; 30)	48	272	19 (10; 28)	79	569	35 (21; 49)
Tomar do Geru	13,536	29	388	28 (18; 39)	54	250	13 (4; 21)	82	887	41 (25; 58)
Japoatã	13,434	37	402	18 (10; 25)	73	305	15 (8; 22)	115	734	33 (19; 46)
Malhador	12,618	33	462	26 (16; 36)	54	275	13 (7; 19)	73	693	40 (24; 56)
Santo Amaro das Brotas	12,102	66	328	16 (6; 26)	73	252	10 (4; 17)	133	580	23 (8; 39)
Gararu	11,604	47	393	23 (12; 34)	71	219	14 (8; 20)	117	600	35 (21; 50)
Moita Bonita	11,335	47	555	24 (12; 37)	65	254	15 (8; 22)	112	672	35 (21; 49)
São Domingos	11,137	44	323	12 (5; 18)	71	249	12 (6; 18)	102	550	27 (14; 40)
Rosário do Catete	10,855	56	379	19 (9; 29)	321	979	53 (28; 79)	389	1,269	70 (35; 105)
Riachuelo	10,213	51	415	20 (11; 29)	96	346	17 (7; 27)	148	738	36 (18; 54)
Arauá	10,056	62	579	29 (18; 40)	70	410	17 (9; 26)	132	816	43 (24; 61)
Pedrinhas	9,602	42	388	23 (14; 33)	68	229	10 (4; 16)	105	558	29 (16; 41)
Pirambu	9,280	61	351	15 (7; 23)	173	474	30 (10; 49)	226	927	51 (28; 75)
Siriri	8,893	58	506	33 (20; 46)	207	449	21 (5; 37)	262	999	56 (27; 84)
Nossa Senhora Aparecida	8,796	34	468	23 (12; 34)	72	268	14 (6; 22)	103	623	32 (18; 45)
Ilha das Flores	8,520	46	493	30 (18; 42)	95	376	17 (7; 26)	129	844	47 (25; 69)
Brejo Grande	8,309	62	448	19 (10; 28)	68	237	11 (4; 18)	142	770	34 (19; 48)
Santana do São Francisco	7,780	56	291	20 (10; 30)	129	551	22 (10; 34)	139	979	47 (26; 67)
Muribeca	7,625	59	622	26 (13; 39)	74	384	17 (9; 25)	132	908	39 (23; 55)
Macambira	6,919	48	481	21 (10; 31)	76	272	15 (6; 24)	123	669	35 (18; 52)
Pinhão	6,576	46	408	21 (12; 29)	99	436	19 (7; 32)	145	796	39 (18; 61)
Nossa Senhora de Lourdes	6,483	23	563	29 (17; 42)	75	361	17 (10; 25)	98	929	45 (28; 61)
Cedro de São João	5,897	60	713	32 (17; 48)	94	340	19 (8; 29)	152	1,042	50 (29; 72)
Graccho Cardoso	5,818	59	532	31 (16; 46)	88	472	21 (10; 31)	148	1,002	48 (23; 73)
Feira Nova	5,584	51	485	27 (16; 39)	82	403	23 (13; 32)	134	876	46 (26; 66)
Divina Pastora	5,138	53	571	29 (17; 42)	293	547	22 (1; 42)	354	1,210	51 (24; 79)
Itabi	4,903	25	436	26 (14; 37)	87	427	23 (12; 34)	112	761	42 (23; 62)
Canhoba	4,008	51	455	25 (15; 35)	114	609	39 (23; 54)	166	1,121	62 (38; 86)
Cumbe	3,987	48	549	32 (19; 45)	125	511	28 (13; 43)	172	1,072	58 (34; 82)
São Miguel do Aleixo	3,930	47	570	30 (17; 44)	168	533	19 (6; 31)	208	1,086	53 (27; 79)
Santa Rosa de Lima	3,913	42	579	24 (14; 35)	129	509	26 (14; 38)	170	981	48 (27; 68)
São Francisco	3,724	64	743	29 (13; 44)	180	584	22 (7; 37)	243	1,223	48 (22; 74)
Malhada dos Bois	3,682	53	718	40 (23; 56)	156	556	33 (17; 49)	210	1,303	71 (42; 100)
General Maynard	3,346	49	448	24 (14; 34)	196	942	42 (22; 61)	242	1,476	70 (40; 100)
Pedra Mole	3,261	53	424	30 (19; 42)	177	756	32 (14; 50)	231	1,290	60 (32; 89)
Telha	3,227	56	599	30 (15; 45)	165	624	32 (17; 46)	221	1,320	64 (34; 95)
Amparo de São Francisco	2,374	59	571	35 (18; 51)	211	873	48 (22; 74)	255	1.452	86 (46; 127)

Rosário do Catete had the highest municipality funded expenditure in 2020, totaling BRL 979.00 per inhabitant. Itabaianinha and Lagarto recorded the lowest municipality funded expenditure in the same year, with BRL 162.00 per inhabitant. All municipalities in Sergipe had an average annual increase in municipality funded expenditure. The highest average annual increase per inhabitant was found in Rosário do Catete, namely BRL 53.00 per inhabitant per year (Table 1). Sixteen municipalities (21.0% of the total) recorded amounts ​​above BRL 1,000.00 per inhabitant in 2020 with regard to total health expenditure. All 75 municipalities showed a statistically significant increase in total health expenditure over the years, with an average of BRL 41.00 per inhabitant (Table 1).

**Figure 2:**
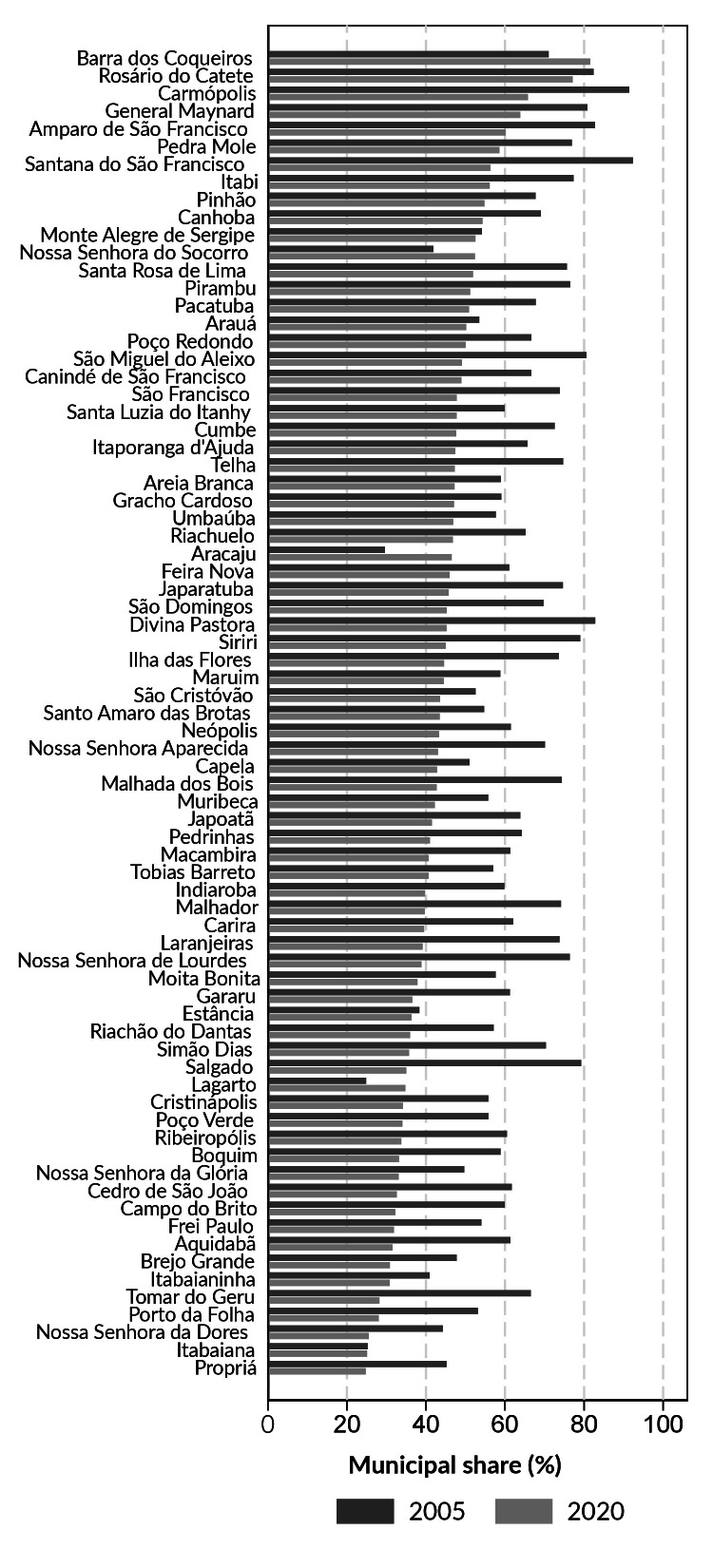
Percentage of total health expenditure of each municipality paid with municipal resources. Sergipe, 2005 and 2020

There was an overall reduction in the percentages of total municipality funded health expenditure between 2005 and 2020, except for Lagarto, Aracaju, Nossa Senhora do Socorro and Barra dos Coqueiros (Figure 2). Barra dos Coqueiros had the highest percentage of total municipality funded health expenditure, exceeding 80.0% in 2020.

**Figure 3:**
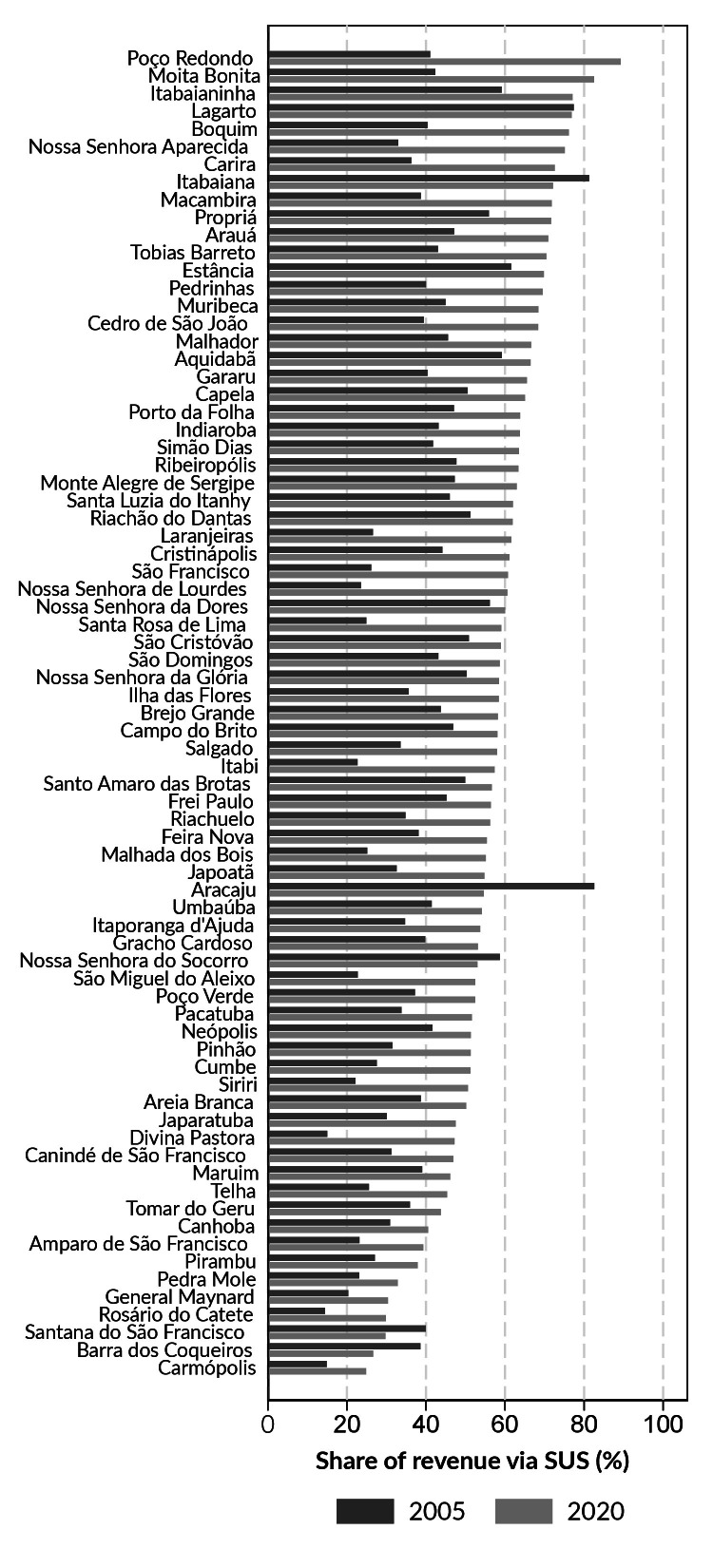
Percentage of total health expenditure of each municipality paid with revenue from Brazilian National Health System funding transfers. Sergipe, 2005 and 2020

There was an increase in the percentage covered by SUS funding transfer revenue over the period, except for Barra dos Coqueiros, Santana do São Francisco, Nossa Senhora do Socorro, Aracaju, Itabaiana and Lagarto (Figure 3).

**Figure 4:**
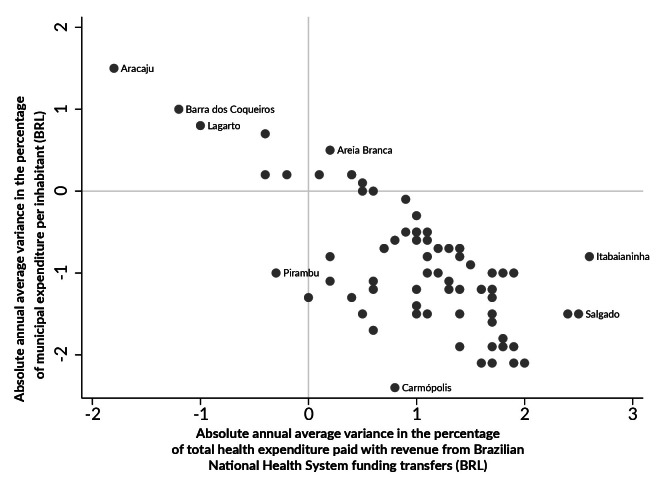
Absolute annual average variance in the percentage of total health expenditure paid with municipal resources and revenue from Brazilian National Health System funding transfers. Sergipe, 2005-2020

We calculated the absolute annual average variance of the percentage of total health expenditure covered by SUS funding transfer revenue and municipality funded expenditure (Figure 4). The municipalities in the first quadrant showed an increase in the shares paid with municipal resources and a reduction in the shares covered by SUS funding transfer revenue. The municipalities in the fourth quadrant had an increase in the shares covered by SUS funding transfer revenue and a reduction in the shares paid with municipal resources, with 80.0% of the municipalities in Sergipe located in this quadrant. Pirambu was the only municipality to register a reduction in both sources of revenue. Four municipalities showed an increase in both indicators, with emphasis on Areia Branca, which had an annual increase of 0.5 and 0.2 in the shares paid with municipal resources and SUS funding transfer revenue, respectively (Figure 4).

## Discussion

This study is the first comprehensive analysis of health funding and expenditure in the 75 municipalities of Sergipe. Variance was observed in SUS health funding transfer revenue among the municipalities, with an average annual increase recorded in all of them. Municipality funded expenditure also showed growth, revealing notable disparities between municipalities. Total health expenditure increased in all municipalities analyzed, with some showing amounts ​​that exceeded the expected limits.

Standing out among the limitations of this study is the use of secondary data sources and the use of aggregated data, these being characteristics inherent to ecological studies. This approach limited the possibility of extrapolating the results to the individual level. Another aspect to be considered was information system weakness, such as the SIOPS system, in providing data on the socioeconomic, political and health impacts on the indicators analyzed. A relevant example was the analysis of the indicators for 2020, a year marked by the beginning of the COVID-19 pandemic. The increases in the indicator amounts recorded that year may have been influenced by the pandemic, since new funding transfer modalities were implemented to face the health crisis (16-18).

We did not analyze revenue from taxation and constitutional transfers to municipalities, which could clarify the availability of municipality funded resources for service managers to implement health policies. Not taking into account funding transfers made by the government of the state of Sergipe to its municipalities made it difficult to assess the degree of dependence of the latter on resources made available by the state.

The analyses revealed discrepancies in the composition of health budgets between the municipalities, especially with regard to the amounts of revenue from SUS health funding transfers. Difficulties faced by the SUS stood out, related to inequalities in the funding pattern adopted by the system (9,10,19).

An analysis of health funding and expenditure in the 26 Brazilian state capital cities found that the amounts per capita of revenue from SUS health funding transfers, in 2018, ranged from BRL 19.00, transferred to Macapá (capital city of the state of Amapá), to BRL 919.00 allocated to Cuiabá (capital city of the state of Mato Grosso) (11). In the state of Bahia, assessment of the distribution of federal financial resources to municipalities in 2010, from the perspective of equity, showed a significant concentration of funds in a limited number of large municipalities (20). At that time, BRL 1.23 billion (60.17% of the total) was transferred to three health macro-regions. The other six macro-regions received 40.0% of the resources, indicating a situation of great unevenness (20).

With a population of 90,072 inhabitants, São Cristóvão had the lowest amount of SUS health funding transfer revenue in 2020. It was the municipality that recorded the lowest average annual increase in this resource, with an increase of BRL 9.00 per inhabitant. São Cristóvão had 26 health centers and invested BRL 200,000.00 of its own resources in the most recent one, covering renovation and acquisition of equipment and furniture (21). In 2020, São Cristóvão’s share in total health care expenditure, funded with municipal resources, was in excess of 40.0% of total expenditure. This fact indicated the true intention of the municipal government to invest in health, even in the face of the low SUS health funding transfer revenue received by the municipality.

Despite the funding difficulties that permeate the SUS, the system continues to be a reference for other public health systems by promoting health guidance, prevention, rehabilitation and maintenance, with a focus on universal and equitable care at different levels of care complexity (22). In the context of health funding, equity is crucial for the development of the SUS, although there are inherent dilemmas in the application of this concept to the public budget (23). Distribution of resources without taking into consideration the principle of equity can compromise care for users of the system who live in conditions of vulnerability, resulting in lack of assistance and restricted access to their constitutional rights (20).

A large difference was found between the highest and lowest amounts of expenditure funded with municipal resources in the municipalities of Sergipe in 2020. The municipality with the highest health expenditure funded with municipal resources was Rosário do Catete (10,855 inhabitants). The lowest expenditures were recorded in Itabaianinha and Lagarto (41,928 and 104,408 inhabitants). A study on municipal public expenditure on health in the 184 municipalities (11 health regions) of the state of Pernambuco, found that the highest municipal public expenditure on health occurred in the third smallest region of the state (24). These findings corroborate our research, by also demonstrating that municipalities and health regions with smaller population sizes had higher health expenditure financed with their own resources than those of more populous municipalities.

All municipalities in Sergipe recorded an average annual increase in expenditure funded with their own resources and in total health expenditure between 2005 and 2020. Considering the pressing need of the Brazilian population for more effective and comprehensive public health actions and services, it is essential that the SUS reaches a new level of expenditure that is compatible with the reduction of regional and sectoral inequalities that still permeate access to and availability of services offered by the system (25).

Regarding total health expenditure, there was a reduction in the percentage of funding with municipal resources between 2005 and 2020, with the exception of four municipalities in the state. Among them, Barra dos Coqueiros stood out in 2020, presenting the highest percentage of share of own resources in total health expenditure. It is worth noting that Barra dos Coqueiros recorded the second highest per capita health expenditure funded with municipal resources, totaling BRL 881.00 per inhabitant.

Analysis of healthcare funding and expenditure in municipalities in the state of São Paulo with a population of over 500,000 inhabitants, with the exception of the state capital, found that the share of funding provided by all seven municipalities studied in total health expenditure was greater than 50.0% (26). For three of those municipalities, their share was in excess of 70.0% (26), which highlighted the lack of a uniform pattern in the funding process for total healthcare expenditure, ranging from large urban centers to municipalities in the country’s smallest state.

Regarding absolute annual average variance of the percentage of total health expenditure covered by revenue from health transfers and funded with municipal resources, Aracaju was one of the few municipalities that showed a reduction in the share of health transfers in the funding of total health expenditure. The results indicated that Aracaju did not suffer an annual reduction in the amount of health transfers; however, the average annual growth of this indicator in the most populous city in Sergipe was lower than the average observed in most municipalities in the state.

The situation of health funding transfers to Aracaju showed divergences in comparison to other municipalities in Sergipe, as well as in relation to other Brazilian state capitals. Individual analyses of health funding and expenditure in Brazilian state capitals revealed that health funding transfer showed a significant annual increase in most capitals. The exceptions were Aracaju and Macapá, where these revenues remained constant between 2008 and 2018, the year in which the analyses were conducted (11). Underfunding of the SUS is one of the main critical points of the Brazilian public health system (27). Over the years, federal health funding transfers have been marked by expenditure containment policies (28). It is important to recognize the historical phase of impasses in the 1990s and 2000s, during which funding stood out as one of the most problematic issues on the health implementation agenda in Brazil (28).

Implementation of the SUS, throughout its existence, has been marked by political and economic tensions (28). Funding of the system is subject to competing forces. On the one hand, emphasis is placed on the “principle of building universality,” which ensures the right of citizens to access health actions and services through the continuous defense of stable financial resources. On the other hand, there is the “principle of expenditure containment,” a defensive strategy guided by economic rationality, in which reduction of public spending is seen as the main instrument to combat the fiscal deficit (28).

It is essential to incorporate a priori health assessments into the policy-making process, such as expenditure containment, so that policies are designed to take public health impacts into account (26). This process requires close collaboration between public health professionals, social scientists, and policy makers (29).

It is recommended that new analyses be conducted to examine healthcare funding and expenditure in other municipalities, states, and health regions, taking into account issues not addressed in this study. Studies of this type are essential to promote systematic monitoring of the sources and applications of resources allocated to healthcare, considering that the SUS is a heritage of all Brazilians and should not be subject to the risk of being scrapped due to the threat of defunding.

This research demonstrated its relevance in addressing aspects related to healthcare budget behavior in the municipalities of Sergipe. The indicators used made it possible to assess the pattern of healthcare funding and expenditure in the state, providing information that can contribute to a more equitable distribution of resources between municipalities, resulting in improvements in the quality of healthcare provided to the population.

We conclude that there was a real increase in revenue from SUS healthcare funding transfers, as well as in expenditure paid for the municipal resources and in total expenditure on health care in all municipalities of Sergipe over the years investigated. However, discrepancies were identified in the distribution of the amounts ​​of revenue from SUS healthcare funding transfers between the municipalities analyzed.

## Data Availability

The database and analysis codes used in this research are available at: https://data.mendeley.com/preview/fnjhkgbvrd?a=1fd0cf06-e44c-499c-8ad3-ee38fe3b0c10.
