# Supplementary material for: Analysis of health funding and expenditure in the municipalities of Sergipe state, Brazil: an ecological study, 2005-2020
Source: Epidemiol Serv Saude. 2025 May 12;34:e20240107. doi: 10.1590/S2237-96222025v34e20240107.en (PMC12077541; doi:10.1590/S2237-96222025v34e20240107.en)
Supplement: Translation

# Translation: Análise do financiamento e dos gastos em saúde nos municípios de Sergipe: estudo ecológico, 2005-2020

**DOI:** 10.1590/S2237-96222025v34e20240107.pt

**Authors:** Millena Barroso Oliveira, Vinícius Henrique Ferreira Pereira de Oliveira, Cauane Blumenberg, Douglas Teixeira da Silva, Álex Moreira Herval, Luiz Renato Paranhos

**Affiliations:** 1 Universidade Federal de Uberlândia, Faculdade de Odontologia, Programa de Pós-Graduação em Odontologia, Uberlândia, MG, Brasil; 2 Universidade Estadual Paulista, Faculdade de Odontologia, Programa de Pós-Graduação em Odontologia, Araraquara, SP, Brasil; 3 Universidade Federal de Pelotas, Faculdade de Medicina, Programa de Pós-Graduação em Epidemiologia, Pelotas, RS, Brasil; 4 Universidade Federal de Uberlândia, Faculdade de Odontologia, Área de Odontologia Preventiva e Social, Uberlândia, MG, Brasil

**Keywords:** Financiamento dos Sistemas de Saúde, Investimentos em Saúde, Gastos em Saúde, Gastos Públicos com Saúde, Estudos Ecológicos

## Abstract

**Objetivo:**

Analisar parte do financiamento e dos gastos em saúde dos municípios de Sergipe.

**Métodos:**

Trata-se de estudo epidemiológico, observacional, do tipo ecológico, com dados do orçamento público em saúde de Sergipe, no período 2005-2020. As unidades de análise foram os municípios do estado. Os dados foram coletados a partir do Sistema de Informações sobre Orçamentos Públicos em Saúde, envolvendo os indicadores: receita das transferências para a saúde – Sistema Único de Saúde, despesa com recursos próprios e despesa total com saúde. As variações anuais médias absolutas dos indicadores de receita e despesa foram estimadas por meio de modelos lineares de mínimos quadrados ponderados para a variância.

**Resultados:**

Foram avaliados os dados de 75 municípios sergipanos. As análises mostraram que, em 2020, o menor valor de receita das transferências para a saúde foi R$ 244,00 por habitante, repassado para São Cristóvão. O maior valor foi R$ 743,00 por habitante, destinado a São Francisco. A receita das transferências para a saúde apresentou aumento em todos os municípios estudados ao longo dos anos analisados, com o aumento anual médio absoluto de R$ 23,00 por habitante.

**Conclusão:**

Observou-se aumento anual médio absoluto na receita das transferências para a saúde destinadas aos municípios. O repasse foi desigual entre os municípios analisados.

## Introdução

O Sistema Único de Saúde (SUS) é reconhecido como um dos maiores sistemas públicos de saúde do mundo, garantindo o acesso universal às ações e serviços de saúde (1-3). Para viabilizar esses objetivos, o financiamento é baseado em um modelo tripartite, no qual a União, os estados e os municípios possuem percentuais mínimos de investimento em saúde (4-6).

Contudo, restrições orçamentárias e desigualdades na distribuição dos recursos comprometem a implementação equitativa do sistema (7). É relevante o acompanhamento sistemático das fontes e do uso dos recursos financeiros efetivamente aplicados em saúde (8). Estudos demonstram que a transferência de recursos federais para a saúde apresenta distribuição desigual entre os estados e municípios, gerando desafios no financiamento da Atenção Primária à Saúde (9,10). 

A análise do financiamento e dos gastos em saúde nas capitais dos estados brasileiros revelou que, entre 2008 e 2018, a transferência de recursos federais para a saúde apresentou padrão de distribuição desigual entre as capitais (11). As análises mostraram que, em 24 das capitais estudadas, houve aumento anual significativo nas receitas de saúde obtidas a partir da transferência de recursos federais. Aracaju foi uma exceção, pois apresentou valor de transferência de recursos federais estatisticamente constante ao longo dos anos analisados (11).

A escassez de análises detalhadas sobre as tendências de financiamento no estado de Sergipe justifica a necessidade de investigação sobre o comportamento dos recursos financeiros destinados à saúde nos municípios sergipanos. Este estudo tem como objetivo analisar parte do financiamento e dos gastos em saúde dos municípios sergipanos.

## Métodos

### Desenho e período do estudo

Trata-se de estudo epidemiológico, observacional, do tipo ecológico. Foi utilizado o guia de reporte REporting of Studies Conducted using Observational Routinely-collected health Data (12) para escrita do trabalho.

Foram utilizados dados do orçamento público em saúde de Sergipe referentes ao período 2005-2020. A amplitude de 15 anos foi escolhida por permitir a análise longitudinal abrangente, viabilizando a observação de tendências e variações nos gastos e receitas ao longo dos anos avaliados. As unidades de análise foram os municípios do estado.

### Local do estudo

Localizado na região Nordeste do Brasil, Sergipe possui área territorial de 21.938,188 km², sendo delimitado a leste pelo oceano Atlântico, a oeste e ao sul pela Bahia e ao norte por Alagoas (Figura 1) (13). O estado tem população estimada em 2.210.004 habitantes. O menor município em termos populacionais em 2020 foi Amparo de São Francisco, com 2.374 habitantes, enquanto Aracaju foi o maior, com 657.013 habitantes. Sergipe conta com 1.115 estabelecimentos de saúde, dos quais 790 são públicos e 325 privados. Entre esses, 921 prestam serviços pelo SUS, totalizando 884 leitos para internação (13).

**Figura 1:** Municípios sergipanos, agrupados de acordo com as três mesorregiões do estado. Sergipe, 2024

### Variáveis

Foram analisados os seguintes indicadores: i) receita das transferências para a saúde – SUS (“R. Transf. SUS”), que abrange as transferências do Ministério da Saúde para o gasto em saúde local (8); ii) despesa com recursos próprios (“D. R. Próprios”), que se refere ao total de despesas com saúde empenhado pelos municípios (8); e iii) despesa total com saúde (“D. Total Saúde”), que representa a despesa total com ações e serviços de saúde, financiadas pelos municípios, estados e governo federal (8).

### Fontes de dados e mensuração

Os dados do orçamento público em saúde dos municípios de Sergipe foram obtidos por meio do Sistema de Informações sobre Orçamentos Públicos em Saúde (Siops), o qual é mantido pelo Ministério da Saúde por meio do Departamento de Informática do SUS (14). Os dados disponíveis na plataforma do Siops são declaratórios e obrigatoriamente informados por cada uma das instâncias do governo. Os indicadores foram coletados como valores brutos em reais para cada um dos anos de interesse. 

Para a coleta de dados, acessou-se a página eletrônica do Siops https://www.gov.br/saude/pt-br/acesso-ainformacao/siops (14). Os ícones “Indicadores” e “Série Histórica de Indicadores (Municípios)” foram selecionados. Em seguida, na página “Indicadores Municipais”, selecionou-se “Sergipe”. Na página seguinte, a coleta de dados compreendeu os indicadores “R. Transf. SUS”, “D. R. Próprios” e “D. Total Saúde”, entre 2005 e 2020. Na aba “Município”, foi selecionada a opção “Todas as categorias”. Todos os dados utilizados no estudo foram obtidos em 24 de janeiro de 2022.

### Deflacionamento

A variação das receitas e despesas ao longo dos anos pode ocorrer simplesmente em virtude da inflação, o que pode comprometer a comparação dos valores ao longo dos diferentes períodos. Visando corrigir esse problema e permitir a comparação de receitas e despesas entre os diferentes anos, os valores foram deflacionados com base no índice nacional de preços ao consumidor amplo (15) de 2020 (último ano considerado na análise).

Para o deflacionamento, a seguinte fórmula foi considerada:

*Valor Real* = *Valor Nominal* × *Índice de Preços*

em que o valor nominal foi o valor em moeda corrente para 2005 de cada um dos três indicadores avaliados. 

Depois de deflacionados, os valores foram utilizados para calcular a razão da receita e despesas pela população residente nos municípios em cada um dos anos – gerando estimativas por habitantes.

### Métodos estatísticos

O primeiro passo da análise estatística consistiu na avaliação da estrutura de covariância dos desfechos de interesse. Modelos de regressões lineares contendo o desfecho de interesse e o ano como preditor foram ajustados para cada município de Sergipe. O teste de Breusch-Pagan (com ajuste de Bonferroni para múltiplas comparações) foi utilizado para avaliar a estrutura da variância e a possibilidade de ocorrência de heterocedasticidade. Nove por cento dos modelos indicaram presença de heterocedasticidade. Optou-se por utilizar modelos lineares de mínimos quadrados ponderados para a variância para estimar as variações anuais médias absolutas da receita das transferências para a saúde – SUS por habitante, despesa com recursos próprios por habitante e despesa total com saúde por habitante. Esses métodos são robustos em situações de heterocedasticidade e permitem estimar adequadamente os coeficientes de regressão, controlando a autocorrelação dos valores de receita e despesa ao longo dos anos. 

O coeficiente gerado por essa regressão pode ser interpretado como a variação anual média absoluta do indicador sendo avaliado, indicando a tendência anual dos indicadores de receita e despesa entre 2005 e 2020. Foram ajustados três modelos de regressão, um para cada indicador de receita e despesa. Nos modelos, os indicadores foram incluídos como desfecho, enquanto o ano (2005-2020) foi considerado como preditor principal. Todas as análises consideraram o nível de significância de 5,0% e foram conduzidas por meio do software Stata 17 (StataCorp LLC., College Station, Texas), utilizando o comando vwls. A significância estatística foi avaliada a partir da sobreposição de estimativas e intervalos de confiança de 95,0%.

## Resultados

Foram avaliados 75 municípios sergipanos. A receita das transferências para a saúde per capita em 2020 variou de R$ 743,00 por habitante, em São Francisco, a R$ 244,00 por habitante, em São Cristóvão. Houve aumento na receita das transferências para a saúde – SUS em todos os 75 municípios analisados, com aumento anual médio de R$ 23,00 por habitante. O maior aumento anual médio foi R$ 40,00 por habitante por ano, registrado em Malhada dos Bois. O menor aumento anual médio foi R$ 9,00 por habitante por ano, observado em São Cristóvão (Tabela 1).

**Tabela 1:** Variação anual média absoluta e intervalo de confiança de 95% (IC95%) da receita das transferências para a saúde – Sistema Único de Saúde por habitante, despesa com recursos próprios por habitante e despesa total com saúde por habitante, por município. Sergipe, 2005 e 2020

Município	População em 2020	Receita das transferências para a saúde – Sistema Único de Saúde (R$)			Despesa com recursos próprios (R$)			Despesa total com saúde (R$)		
		2005	2020	Variação (IC95%)	2005	2020	Variação (IC95%)	2005	2020	Variação (IC95%)
Aracaju	657.013	194	478	15 (4; 26)	70	407	25 (16; 35)	235	875	42 (22; 62)
Nossa Senhora do Socorro	183.628	60	288	12 (5; 20)	43	285	13 (7; 20)	103	543	24 (13; 36)
Lagarto	104.408	84	359	14 (8; 20)	27	162	10 (6; 14)	108	466	23 (15; 32)
Itabaiana	95.427	86	539	26 (15; 37)	27	187	11 (7; 15)	106	747	38 (24; 52)
São Cristóvão	90.072	30	244	9 (5; 14)	31	180	9 (5; 13)	60	414	19 (11; 27)
Estância	69.184	96	469	24 (12; 36)	59	244	14 (8; 21)	155	672	36 (20; 52)
Tobias Barreto	52.191	35	296	14 (8; 20)	47	171	8 (3; 13)	82	420	22 (12; 32)
Itabaianinha	41.928	33	404	34 (6; 62)	23	162	9 (5; 13)	55	525	31 (20; 43)
Simão Dias	40.484	26	332	20 (13; 28)	43	186	10 (6; 14)	61	522	32 (19; 44)
Nossa Senhora da Glória	36.924	37	377	22 (11; 33)	37	213	12 (7; 16)	74	644	31 (18; 44)
Poço Redondo	34.775	39	300	17 (10; 24)	63	168	10 (5; 14)	95	336	26 (13; 38)
Itaporanga d’Ajuda	34.356	30	442	24 (15; 33)	56	390	21 (13; 28)	86	824	44 (29; 60)
Capela	34.213	36	402	20 (12; 28)	36	265	11 (5; 17)	71	618	28 (16; 39)
Barra dos Coqueiros	30.407	45	288	12 (8; 17)	82	881	47 (31; 64)	116	1.080	60 (40; 80)
Canindé de São Francisco	29.900	67	412	24 (13; 34)	144	430	6 (-4; 16)	216	879	33 (16; 50)
Laranjeiras	29.826	49	426	21 (13; 30)	136	271	19 (4; 35)	184	692	40 (22; 59)
Propriá	29.626	49	576	34 (22; 47)	39	199	11 (6; 16)	87	804	46 (32; 59)
Porto da Folha	28.596	33	436	26 (16; 36)	38	192	10 (6; 14)	71	683	35 (23; 47)
Boquim	26.816	33	410	21 (12; 29)	49	178	9 (5; 13)	83	538	28 (17; 40)
Nossa Senhora da Dores	26.629	50	554	29 (17; 40)	39	236	16 (7; 25)	89	924	49 (30; 68)
Umbaúba	25.294	35	278	15 (8; 23)	49	241	13 (7; 19)	86	514	27 (16; 38)
Poço Verde	23.728	36	315	17 (10; 24)	55	204	11 (5; 17)	98	600	30 (17; 43)
Carira	22.082	28	393	20 (13; 28)	47	214	10 (5; 15)	76	542	30 (19; 40)
Aquidabã	21.563	46	422	21 (13; 30)	47	201	10 (5; 15)	77	635	34 (21; 46)
Salgado	19.998	29	320	23 (14; 32)	68	194	9 (5; 13)	85	552	27 (17; 38)
Riachão do Dantas	19.805	36	358	19 (12; 26)	40	208	8 (3; 12)	71	578	26 (16; 36)
Campo do Brito	18.109	40	387	24 (13; 34)	51	215	13 (5; 22)	84	666	36 (23; 50)
Japaratuba	18.743	55	449	23 (10; 37)	138	432	17 (6; 28)	184	945	44 (22; 65)
Neópolis	18.719	40	354	22 (13; 31)	58	299	15 (9; 22)	95	691	42 (26; 58)
Ribeiropólis	18.652	46	466	23 (15; 32)	58	248	14 (8; 21)	96	735	35 (23; 47)
Areia Branca	18.542	36	400	18 (10; 27)	55	377	22 (12; 32)	94	798	38 (22; 55)
Indiaroba	17.957	49	399	17 (8; 25)	68	249	12 (6; 18)	113	626	33 (18; 48)
Cristinápolis	17.874	37	490	27 (18; 37)	46	274	18 (10; 25)	83	802	45 (31; 59)
Maruim	17.213	55	409	25 (16; 33)	83	394	23 (14; 33)	142	886	49 (32; 67)
Carmópolis	16.634	26	288	16 (9; 24)	162	764	25 (11; 40)	178	1.161	53 (34; 71)
Frei Paulo	15.421	45	416	22 (13; 32)	54	235	13 (6; 20)	100	737	35 (21; 48)
Monte Alegre de Sergipe	15.031	47	379	19 (9; 28)	54	316	16 (11; 21)	99	601	31 (19; 43)
Pacatuba	14.428	57	366	24 (15; 32)	114	361	19 (10; 28)	168	710	38 (24; 53)
Santa Luzia do Itanhy	14.035	36	353	19 (9; 30)	48	272	19 (10; 28)	79	569	35 (21; 49)
Tomar do Geru	13.536	29	388	28 (18; 39)	54	250	13 (4; 21)	82	887	41 (25; 58)
Japoatã	13.434	37	402	18 (10; 25)	73	305	15 (8; 22)	115	734	33 (19; 46)
Malhador	12.618	33	462	26 (16; 36)	54	275	13 (7; 19)	73	693	40 (24; 56)
Santo Amaro das Brotas	12.102	66	328	16 (6; 26)	73	252	10 (4; 17)	133	580	23 (8; 39)
Gararu	11.604	47	393	23 (12; 34)	71	219	14 (8; 20)	117	600	35 (21; 50)
Moita Bonita	11.335	47	555	24 (12; 37)	65	254	15 (8; 22)	112	672	35 (21; 49)
São Domingos	11.137	44	323	12 (5; 18)	71	249	12 (6; 18)	102	550	27 (14; 40)
Rosário do Catete	10.855	56	379	19 (9; 29)	321	979	53 (28; 79)	389	1.269	70 (35; 105)
Riachuelo	10.213	51	415	20 (11; 29)	96	346	17 (7; 27)	148	738	36 (18; 54)
Arauá	10.056	62	579	29 (18; 40)	70	410	17 (9; 26)	132	816	43 (24; 61)
Pedrinhas	9.602	42	388	23 (14; 33)	68	229	10 (4; 16)	105	558	29 (16; 41)
Pirambu	9.280	61	351	15 (7; 23)	173	474	30 (10; 49)	226	927	51 (28; 75)
Siriri	8.893	58	506	33 (20; 46)	207	449	21 (5; 37)	262	999	56 (27; 84)
Nossa Senhora Aparecida	8.796	34	468	23 (12; 34)	72	268	14 (6; 22)	103	623	32 (18; 45)
Ilha das Flores	8.520	46	493	30 (18; 42)	95	376	17 (7; 26)	129	844	47 (25; 69)
Brejo Grande	8.309	62	448	19 (10; 28)	68	237	11 (4; 18)	142	770	34 (19; 48)
Santana do São Francisco	7.780	56	291	20 (10; 30)	129	551	22 (10; 34)	139	979	47 (26; 67)
Muribeca	7.625	59	622	26 (13; 39)	74	384	17 (9; 25)	132	908	39 (23; 55)
Macambira	6.919	48	481	21 (10; 31)	76	272	15 (6; 24)	123	669	35 (18; 52)
Pinhão	6.576	46	408	21 (12; 29)	99	436	19 (7; 32)	145	796	39 (18; 61)
Nossa Senhora de Lourdes	6.483	23	563	29 (17; 42)	75	361	17 (10; 25)	98	929	45 (28; 61)
Cedro de São João	5.897	60	713	32 (17; 48)	94	340	19 (8; 29)	152	1.042	50 (29; 72)
Graccho Cardoso	5.818	59	532	31 (16; 46)	88	472	21 (10; 31)	148	1.002	48 (23; 73)
Feira Nova	5.584	51	485	27 (16; 39)	82	403	23 (13; 32)	134	876	46 (26; 66)
Divina Pastora	5.138	53	571	29 (17; 42)	293	547	22 (1; 42)	354	1.210	51 (24; 79)
Itabi	4.903	25	436	26 (14; 37)	87	427	23 (12; 34)	112	761	42 (23; 62)
Canhoba	4.008	51	455	25 (15; 35)	114	609	39 (23; 54)	166	1.121	62 (38; 86)
Cumbe	3.987	48	549	32 (19; 45)	125	511	28 (13; 43)	172	1.072	58 (34; 82)
São Miguel do Aleixo	3.930	47	570	30 (17; 44)	168	533	19 (6; 31)	208	1.086	53 (27; 79)
Santa Rosa de Lima	3.913	42	579	24 (14; 35)	129	509	26 (14; 38)	170	981	48 (27; 68)
São Francisco	3.724	64	743	29 (13; 44)	180	584	22 (7; 37)	243	1.223	48 (22; 74)
Malhada dos Bois	3.682	53	718	40 (23; 56)	156	556	33 (17; 49)	210	1.303	71 (42; 100)
General Maynard	3.346	49	448	24 (14; 34)	196	942	42 (22; 61)	242	1.476	70 (40; 100)
Pedra Mole	3.261	53	424	30 (19; 42)	177	756	32 (14; 50)	231	1.290	60 (32; 89)
Telha	3.227	56	599	30 (15; 45)	165	624	32 (17; 46)	221	1.320	64 (34; 95)
Amparo de São Francisco	2.374	59	571	35 (18; 51)	211	873	48 (22; 74)	255	1.452	86 (46; 127)

Rosário do Catete apresentou a maior despesa com recursos próprios em 2020, totalizando R$ 979,00 por habitante. Itabaianinha e Lagarto registraram a menor despesa com recursos próprios no mesmo ano, com R$ 162,00 por habitante. Todos os municípios de Sergipe tiveram aumento anual médio na despesa com recursos próprios. O maior aumento anual médio por habitante foi observado em Rosário do Catete, com valor de R$ 53,00 por habitante por ano (Tabela 1).

Dezesseis municípios (21,0% do total) registraram valores superiores a R$ 1.000,00 por habitante em 2020 em relação à despesa total com saúde. Todos os 75 municípios apresentaram aumento estatisticamente significativo na despesa total com saúde ao longo dos anos, com média de R$ 41,00 por habitante (Tabela 1).

Observou-se redução geral nos percentuais da despesa total com saúde custeados com recursos municipais entre 2005 e 2020, exceto para Lagarto, Aracaju, Nossa Senhora do Socorro e Barra dos Coqueiros (Figura 2). Barra dos Coqueiros apresentou o maior percentual da despesa total com saúde custeado com recursos próprios, ultrapassando 80,0% em 2020.

**Figura 2:** Percentual da despesa total com saúde de cada município, paga com recursos municipais. Sergipe, 2005 e 2020

Houve aumento no percentual coberto por transferências para a saúde ao longo do período, exceto para Barra dos Coqueiros, Santana do São Francisco, Nossa Senhora do Socorro, Aracaju, Itabaiana e Lagarto (Figura 3).

**Figura 3:** Percentual da despesa total com saúde de cada município, paga pela receita das transferências para a saúde – Sistema Único de Saúde (SUS). Sergipe, 2005 e 2020

A variação anual média absoluta do percentual da despesa total com saúde coberta pela receita das transferências para a saúde e pelos recursos municipais foi calculada (Figura 4). Os municípios no primeiro quadrante apresentaram aumento nas quotas pagas com recursos municipais e redução nas quotas cobertas por receita das transferências para a saúde – SUS. Os municípios localizados no quarto quadrante tiveram aumento nas quotas cobertas por receita das transferências para a saúde – SUS e redução nas quotas pagas com recursos municipais, com 80,0% dos municípios de Sergipe localizados nesse quadrante. Pirambu foi o único município a registrar redução em ambas as grandezas. Quatro municípios apresentaram aumento em ambos os indicadores, com destaque para Areia Branca, que teve aumento anual de 0,5 e 0,2 nas quotas pagas com recursos municipais e receita das transferências para a saúde – SUS (Figura 4).

**Figura 4:** Variação anual média absoluta do percentual da despesa total com saúde coberta com recursos municipais e receita das transferências para a saúde – Sistema Único de Saúde (SUS). Sergipe, 2005-2020

## Discussão

Este estudo representa a primeira análise abrangente do financiamento e dos gastos em saúde nos 75 municípios de Sergipe. Foi observada variação na receita das transferências para a saúde entre os municípios, com aumento anual médio registrado em todos eles. A despesa com recursos próprios também demonstrou crescimento, evidenciando disparidades notáveis entre os municípios. A despesa total com saúde aumentou em todos os municípios analisados, com alguns apresentando valores que ultrapassaram os limites esperados.

Entre as limitações do estudo, destacaram-se a utilização de fontes secundárias de dados e o emprego de dados agregados, características inerentes aos estudos ecológicos. Essa abordagem limitou a possibilidade de extrapolar os resultados para o nível individual. Outro aspecto a ser considerado foi a fragilidade dos sistemas de informação, como o Siops, na disponibilização de dados sobre os impactos socioeconômicos, políticos e sanitários nos indicadores analisados. Um exemplo relevante foi a análise dos indicadores para 2020, ano marcado pelo início da pandemia de covid-19. Os aumentos nos valores dos indicadores registrados nesse ano podem ter sido influenciados pela pandemia, uma vez que novas modalidades de transferências foram implementadas para enfrentar a crise sanitária (16-18).

Não foi realizada análise da receita proveniente de impostos e transferências constitucionais dos municípios, o que poderia esclarecer a disponibilidade de recursos próprios para os gestores municipais na implementação de políticas de saúde. A falta de consideração das transferências de Sergipe para seus municípios dificultou a avaliação do grau de dependência destes em relação aos recursos disponibilizados pelo estado.

As análises revelaram discrepâncias na composição dos orçamentos de saúde entre os municípios, especialmente no que se refere aos valores das receitas de transferências para a saúde – SUS. Foram apontadas dificuldades enfrentadas pelo SUS, relacionadas às desigualdades no padrão de financiamento adotado pelo sistema (9,10,19).

Em uma análise do financiamento e do gasto em saúde nas 26 capitais dos estados brasileiros, observou-se que os valores da receita das transferências para a saúde – SUS per capita, em 2018, variaram de R$ 19,00, transferidos para Macapá, Amapá, a R$ 919,00 destinados a Cuiabá, Mato Grosso (11). Na Bahia, a avaliação da distribuição dos recursos financeiros federais para os municípios, em 2010, sob a perspectiva da equidade, evidenciou significativa concentração de verbas em um número restrito de municípios de grande porte (20). Na ocasião, R$ 1,23 bilhão (60,17% do total) foi repassado para três macrorregiões de saúde. As demais seis macrorregiões receberam 40,0% dos recursos, indicando uma situação de grande desequilíbrio (20).

Com população de 90.072 habitantes, São Cristóvão apresentou o menor valor de repasse de receita das transferências para a saúde – SUS em 2020. Foi o município que registrou o menor aumento anual médio desse recurso, com incremento de R$ 9,00 por habitante. São Cristóvão contava com 26 unidades de saúde. Para a mais recente, foram investidos R$ 200 mil em recursos próprios, abrangendo reforma e aquisição de equipamentos e mobiliário (21). Em 2020, a participação de São Cristóvão na despesa total com saúde, financiada por recursos municipais, ultrapassou 40,0% do total das despesas. Esse fato indicou a real intenção do governo municipal em investir em saúde, mesmo diante da baixa receita das transferências para a saúde – SUS recebida pelo município.

Apesar das dificuldades de financiamento que permeiam o SUS, o sistema continua sendo referência para outros sistemas públicos de saúde ao promover orientação, prevenção, reabilitação e manutenção da saúde, com foco no atendimento universal e equitativo em diferentes níveis de complexidade da atenção (22). No contexto do financiamento da saúde, a equidade é crucial para o desenvolvimento do SUS, embora existam dilemas inerentes à aplicação desse conceito no orçamento público (23). A distribuição dos recursos sem considerar o princípio da equidade pode comprometer a atenção aos usuários do sistema que vivem em condições de vulnerabilidade, resultando em desassistência e restrição ao acesso a seus direitos constitucionais (20).

Foi vista grande diferença entre os valores da maior e da menor despesa com recursos próprios nos municípios de Sergipe em 2020. O município com a maior despesa em saúde financiadas por recursos municipais foi Rosário do Catete (10.855 habitantes). As menores despesas foram registradas em Itabaianinha e Lagarto (41.928 e 104.408 habitantes). Considerando a despesa pública municipal com saúde nos 184 municípios (11 regiões de saúde) de Pernambuco, foi constatado que a maior despesa pública municipal em saúde ocorreu na terceira menor região do estado (24). Esses achados corroboram esta pesquisa, ao também demonstrar que municípios e regiões de saúde com menor porte populacional apresentaram despesas em saúde financiadas com recursos próprios superiores às de municípios mais populosos.

Todos os municípios de Sergipe registraram aumento anual médio nas despesas com recursos próprios e na despesa total com saúde entre 2005 e 2020. Considerando a necessidade premente da população brasileira por ações e serviços públicos de saúde mais eficazes e abrangentes, é fundamental que o SUS atinja novo patamar de gastos que seja compatível com a redução das desigualdades regionais e setoriais que ainda permeiam o acesso e a disponibilidade dos serviços oferecidos pelo sistema (25).

Quanto à despesa total com saúde, observou-se redução no percentual de financiamento com recursos municipais entre 2005 e 2020, com exceção de quatro municípios do estado. Entre eles, Barra dos Coqueiros se destacou em 2020, apresentando o maior percentual de participação de recursos próprios sobre a despesa total com saúde. Vale destacar que Barra dos Coqueiros registrou a segunda maior despesa per capita em saúde custeada com recursos municipais, totalizando R$ 881,00 por habitante.

Em uma análise do financiamento e gasto com saúde nos municípios paulistas com população superior a 500 mil habitantes, excetuando a capital, observou-se que todos os sete municípios estudados apresentaram percentual de participação com recursos próprios sobre as despesas totais superior a 50,0% (26). Em três desses municípios, esse percentual ultrapassou 70,0% (26), o que evidenciou a ausência de padrão uniforme no processo de financiamento da despesa total com saúde, variando desde grandes centros urbanos até municípios do menor estado do país.

Na variação anual média absoluta do percentual da despesa total com saúde coberta pela receita das transferências para a saúde e custeadas com recursos municipais, Aracaju foi um dos poucos municípios que apresentaram redução na participação das transferências para a saúde no financiamento da despesa total com saúde. Os resultados indicaram que Aracaju não sofreu redução anual no montante das transferências para a saúde; porém, o crescimento anual médio desse indicador na cidade mais populosa de Sergipe foi inferior à média observada na maioria dos municípios do estado.

A situação do repasse das receitas de transferências para a saúde para Aracaju apresentou divergências em comparação aos demais municípios de Sergipe, bem como em relação às outras capitais brasileiras. Análises individuais do financiamento e dos gastos em saúde das capitais dos estados brasileiros revelaram que as receitas de transferências para a saúde apresentaram aumento anual significativo na maioria das capitais. As exceções foram Aracaju e Macapá, onde essas receitas permaneceram constantes entre 2008 e 2018, ano em que as análises foram realizadas (11). O subfinanciamento do SUS constitui um dos principais pontos críticos do sistema público de saúde brasileiro (27). Ao longo dos anos, os repasses federais para a saúde têm sido marcados por políticas de contenção de gastos (28). É importante reconhecer a fase histórica de impasses nas décadas de 1990 e 2000, durante a qual o financiamento se destacou como um dos temas mais problemáticos na agenda de implementação da saúde no Brasil (28).

A implementação do SUS, ao longo de sua existência, tem sido marcada por tensões políticas e econômicas (28). O financiamento do sistema segue um duplo movimento. Por um lado, enfatiza-se o “princípio da construção da universalidade”, que assegura o direito à cidadania no acesso às ações e aos serviços de saúde, por meio da defesa contínua de recursos financeiros estáveis. Por outro lado, existe o “princípio da contenção de gastos”, estratégia defensiva orientada pela racionalidade econômica, na qual a redução das despesas públicas é vista como o principal instrumento para combater o déficit fiscal (28).

É fundamental incorporar avaliações de saúde a priori no processo de formulação de políticas, como as de contenção de gastos, para que essas políticas sejam planejadas de maneira a considerar os impactos na saúde pública (26). Esse processo requer colaboração estreita entre profissionais de saúde pública, cientistas sociais e formuladores de políticas (29).

Recomenda-se a realização de novas análises que examinem o financiamento e o gasto com saúde em outros municípios, estados e regiões de saúde, levando em consideração as questões não abordadas neste estudo. Estudos desse tipo são fundamentais para promover o acompanhamento sistemático das fontes e do uso dos recursos destinados à saúde, considerando que o SUS é um patrimônio de todos os brasileiros e não deve estar sujeito ao risco de sucateamento em decorrência da ameaça de desfinanciamento.

Esta pesquisa demonstrou sua relevância ao abordar aspectos relacionados ao comportamento orçamentário em saúde nos municípios de Sergipe. Os indicadores utilizados possibilitaram a avaliação do padrão de financiamento e gasto com saúde no estado, fornecendo informações que podem contribuir para a distribuição mais equitativa dos recursos entre os municípios, o que resulta em melhorias na qualidade da assistência à saúde prestada à população.

Conclui-se que houve aumento real na receita de transferências para a saúde – SUS, na despesa com recursos próprios e na despesa total com saúde em todos os municípios de Sergipe ao longo dos anos investigados. Contudo, foram identificadas discrepâncias na distribuição dos valores da receita de transferências para a saúde – SUS entre os municípios analisados.

## Data Availability

O banco de dados e os códigos de análise utilizados na pesquisa estão disponíveis em: https://data.mendeley.com/preview/fnjhkgbvrd?a=1fd0cf06-e44c-499c-8ad3-ee38fe3b0c10.
